# Mortality of *Phyllophaga vetula* larvae by the separate and combined application of *Metarhizium anisopliae*, *Steinernema carpocapsae* and *Steinernema glaseri*

**DOI:** 10.21307/jofnem-2020-068

**Published:** 2020-07-29

**Authors:** Jaime Ruiz-Vega, Carlos I. Cortés-Martínez, Teodulfo Aquino-Bolaños, Pastor T. Matadamas-Ortíz, Cipriano García-Gutiérrez, José Navarro-Antonio

**Affiliations:** 1 Instituto Politécnico Nacional, CIIDIR U. Oaxaca, Protección y Producción Vegetal, Santa Cruz Xoxocotlán, Oaxaca 71230, México; 2 Cuerpo Académico de Biotecnología Agroalimentaria, Instituto de Ciencias Agropecuarias, Universidad Autónoma del Estado de Hidalgo, Tulancingo de Bravo, Hidalgo 43600, México; 3 Instituto Politécnico Nacional, CIIDIR U. Sinaloa, Bulevard Juan de Dios Bátiz Paredes 250, Colonia San Joachin, Guasave, Sinaloa, CP 81101, México

**Keywords:** Entomopathogenic fungi, Entomopathogenic nematodes, Mixed infection, White grub, *Zea mays*

## Abstract

*Phyllophaga* spp. are a complex of edaphic insect pests that are present in the corn crops (*Zea mays*) in México, which are usually controlled with increasing dosages of broad-spectrum chemical insecticides. Several entomopathogenic nematode species can produce acceptable control levels of these larvae. However, the synergistic interaction between fungi and entomopathogenic nematodes (EPN) could improve the control of this insect. This study investigates the mortality of larvae of *Phyllophaga vetula* by the effect of the separate or combined application of the fungus *Metarhizium anisopliae* M1cog strain (Ma) and the nematodes *Steinernema carpocapsae* All strain (Sc) or *Steinernema glaseri* NJ-43 strain (Sg). In laboratory, dosages of 1 × 10^6^ or 1 × 10^8^ spores/larva and 250 infective juveniles were applied on medium or large size *P. vetula* larvae contained in vials with sterilized agricultural soil as the assay arena. The separate application of Ma did not kill any larvae, but Sg and Sc killed 40 and 80% of the larvae, respectively. However, the Ma and Sc combination had an important antagonistic interaction that decreased the mortality to 40%, but the combination Ma and Sg had a slight additive interaction that increased the mortality to 47%. The most determining factor in larvae mortality was the nematode used, with Sg as the species with best performance in 6 of the 12 treatments evaluated and with a maximum effectivity of 80% on medium-size larvae if combined with a low dosage of Ma. The combined application of an entomopathogenic fungus and EPN showed no consistent effects on the mortality percentage of *P. vetula*, mostly because the fungus was not isolated from *Phyllophaga* larvae.

White grubs of *Phyllophaga* spp. (Coleoptera: Scarabaeidae) are the principal soil-living pests in corn production (*Zea mays*), affecting seed quality and yield (Jackson and Klein, 2006). In corn fields of the central valleys of Oaxaca, Mexico, one of the most abundant species is *P. vetula* (Horn), which causes yield loses up to 50% depending on plant stand losses (Ruiz-Vega et al., 2003). In Central Mexico, a yield loss of 500 to 1,400 kg.ha−1 is expected (Villalobos, 1992). However, the use of chemical pesticides for the control of white grubs is causing increased resistance of this insect pest and has resulted in negative effects on the environment and human health, which make necessary the development of effective biological control products with low impact on the environment and human health (Cory and Franklin, 2012; Chandel et al., 2019; Karabörklü et al., 2018).

The combined application of entomopathogenic fungi (EPF) and entomopathogenic nematodes (EPN) through a formulated product is an alternative solution that is being tested to improve the effectiveness of the microbial pesticides used in integrated pest management programs (Wakil et al., 2017). It is believed that by the action mode of EPF, their previous application on the insect hosts increases their susceptibility to EPN infection (Ansari et al., 2008; Shaurub et al., 2016). However, in combination with nematophagous fungi, a reduction in the EPN and EPF activity has been reported (Bueno-Pallero et al., 2018).

The first challenge in the development of these products is to get a combination of entomopathogenic agents (EAs) that favor a synergist or additive interaction in order to achieve an effective control because biological, environmental, and management factors influence the performance of EAs.

The separate application of the entomopathogens *Metarhizium* spp., *Heterorhabditis* spp., and *Steinernema* spp. usually shows a good percentage of control over *Phyllophaga* spp., an edaphic pest that feeds on roots, decreases the yield and quality of the corn-crop *Zea mays* (Jackson and Klein, 2006; Gassmann and Clifton, 2017), but in Mexico *Phyllophaga* spp. is usually controlled with broad-spectrum chemical insecticides such as carburofan and heptachlor (Ruiz-Vega et al., 2003).

*S. glaseri* (NJ-43 strain) is a nematode of greater effectiveness than *S. carpocapsae* (All strain) against some species of *Phyllophaga* spp. (Ruiz-Vega et al., 2003; Girón-Pablo et al., 2015; Cortés-Martínez et al., 2017), while *S. carpocapsae* had an intermediate performance because it required four days to control 50% of the larvae when applied at doses of 200 IJs (Ruiz-Vega et al., 2003). The fungus *M. anisopliae* (HI-019) is more effective than *Bauveria bassiana*, killing up to 86% of *P. vetula* larvae treated with 1 × 10^8^ conidia.mL−1 after 12 days post-inoculation (Pérez et al., 2016).

In laboratory, additive interactions have been observed between *H. megidis* and *B. bassiana*, and between *H. bacteriophora* and *B. bassiana* or *M. anisopliae* in most of the combinations applied on *Cyclocephala lurida* (Coleoptera: Scarabaeidae). However, the combined effect was not significantly different for simultaneous applications of nematodes or fungi or carried out at different times (Wu et al., 2014). In a study in laboratory and greenhouse conditions, where the interaction between four EPN and two EPFs was evaluated, synergies were found between nematode species applied after the fungi at different times, while the separate application of *B. bassiana* and *M. anisopliae* did not reduce the population of third-stage larvae of *Cyclocephala lurida* Bland (Martínez-Hernández et al., 2015).

Except for a few studies, the effect of the combined application of certain species such as *Metarhizium* spp. and *Steinernema* spp. on *Phyllophaga* spp. is still little explored and could result in a synergistic or additive effect with better effectiveness than applied separately. Therefore, the aim of this work was to evaluate the effect of separate and combined application of the fungus *M. anisopliae* and the nematode *S. glaseri* or *S. carpocapsae* on the mortality of *P. vetula* and to determine its type of interaction in a four-day deferred application between EAs.

## Materials and methods

### *P. vetula* larvae

Larvae of *P. vetula* were collected from two different corn-crop fields in the village of Cuilapam de Guerrero, Oaxaca, Mexico (17° 00´ N, 96° 46´ W, 1596 masl) in July, 2019. The presence of larvae in the earlier stages of growth of corn plants (*Zea mays*) and the appearance of visible damage in the plants were the criteria for selecting the sampling site. Each larva was collected manually from a 0 to 20 soil depth and confined individually in a 50 mL cylindrical plastic container that was filled with sterilized agricultural soil from the collection site and a slice of carrot as feed. In laboratory, the *P. vetula* larvae were classified by their size as medium (1.8 cm < Me < 2.5 cm) or as large (2.5 cm < La ≥ 3.2 cm) and kept at room temperature (23 ± 6°C). During a week, insects were observed in order to separate the dead, injured, or infected larvae.

### Entomopathogenic agents

The EPF used was *M. anisopliae* M1cog strain (Ma) Access GenBank KR998522 (Metchnikoff) Sorokin (1883), which was isolated from *Spodoptera frugiperda*, was provided by the “Laboratorio de Bioinsecticidas of CIIDIR IPN Unidad Sinaloa.” The nematodes *Steinernema carpocapsae* All strain (Sc) Access GenBank CM016762.1 (Weiser, 1955) and *Steinernema glaseri* NJ-43 strain (Sg) Access GenBank AF122015.1 (Steiner, 1929) are from the collection of the “Laboratorio de Nematodos Entomopatógenos of CIIDIR IPN Unidad Oaxaca,” which was initiated with nematode species donated by the EPN Laboratory of the University of California, Davis. The nematodes were reproduced using third-instar larvae of the wax moth *Galleria mellonella* (Kaya and Stock, 1997) and stored at 12°C for 15 days before use.

### Infectivity assay

Infectivity, defined as the proportion of dead insects after being exposed to entomopathogens (Ricci et al., 1996), was tested every seven days from the moment of application of Ma. Eight observation periods were completed, i.e., for a total of 56 days. The assay arena was a 100 mL cylindrical container filled with loamy-sand soil and a larva of size Me or La. *P. vetula* larvae were inoculated first with Ma in high (H) or low (L) doses, 1 × 10^8^ and 1 × 10^6^ spores/insect, respectively. Four days later, the nematodes Sg or Sc were applied at a dose of 250 IJs/insect (20 IJs/cm−1) on the surface of the arena. This apparently large amount was applied to ensure infection because the larvae were already buried into the soil. The dilution procedure proposed by Stock and Goodrich-Blair (2012) was followed to estimate the IJ’s concentration.

Insect mortality was determined when the larvae did not move when stimulated with a dissecting needle. To confirm the cause of death, each cadaver presenting signs of infection was transferred to a petri dish lined with a filter paper disc (90 mm in diameter, Whatman No. 1) and incubated at 23 ± 2°C until the appearance of spores of Ma (indicative of fungal infection) or the emergence of IJs (indicative of infection by nematodes) happened.

### Experimental design and treatments

To evaluate the effect of the application of fungus Ma and the nematodes Sc and Sg on the mortality of *P. vetula*, two sizes of larvae were inoculated in a multifactorial experiment 2^3^, where the factors were: (i) size of the larva (Me or La), (ii) dose of Ma (H or L), and (iii) species of EPN (Sc or Sg). The eight resulting treatments were: LaHSc, LaHSg, LaLSc, LaLSg, MeHSc, MeHSg, MeLSc, and MeLSg. One larva of size Me or La was placed in each of 15 containers filled with field-capacity moist agricultural soil per treatment to determine insect mortality, including the control treatment. The experiment was conducted twice. The treatments LaH, LaSg, MeL, and MeSc were included to evaluate the type of interaction on the LaHSg and MeLSc treatments and the effect of increasing the dose of Ma in larvae of size Me or La.

### Determination of the interaction between *M. anisopliae* and nematodes on *P. vetula*

The type of interaction (synergistic or antagonistic) between EPF and EPN was determined in the combined treatment LaHSg considering LaH and LaSg effects, whereas for the treatment MeLSc, the effects of MeL and MeSc were considered. The expected mortality *E*_*m*_ in the combined application was estimated using the formula *E*_*m*_ = *M*_*n*_ + *M*_*f*_ (1−*M*_*n*_), where *M*_*n*_ and *M*_*f*_ are the mortality percentage observed for nematodes and fungi inoculated separately, respectively. Then, the $\chi_{e}^{2}$ test was carried out with the estimated value of observed mortality *M*_*n+f*_ in combined applications and compared with the value in tables of $\chi_{t}^{2}$ for 1 degree of freedom, with *p* < 0.05. According to the criterion established, if it is fulfilled that $\chi_{t}^{2}>\chi_{e}^{2}$, the interaction is additive, or it is synergistic/additive if that criterion is not met. Then, if the difference between *M*_*n+f*_ and *E*_*m*_ has a positive or negative value, it is considered that there is a synergistic or antagonistic interaction, respectively (Mcvay et al., 1977).

### Statistical analysis of data

The accumulated mortality per treatment after 56 days was used to estimate the mortality percentage, i.e. the number of dead larvae in proportion to the number of larvae that was used. The data were transformed into ranges to satisfy the normality criteria. The effect of the factors of insect size, dose of Ma and EPN species on mortality percentage was determined by using a factorial analysis of variance (ANOVA) and applying the Tukey test (*p* < 0.05). Also, a Pearson correlation analysis was performed to determine the relationship between increasing doses of Ma before the application of Sg or Sc and the mortality percentage of larvae of size Me or La (*p* < 0.05). All analyzes were performed on SigmaPlot^®^ 12 (Systat Software, Inc., San José, CA, USA).

## Results

### Interaction between fungi and EPN on *P. vetula* larvae

**Figure 1:**
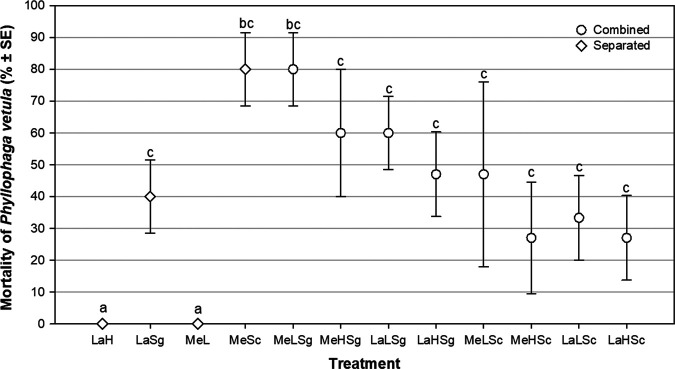
Mortality of medium (Me) or large (La) *Phyllophaga vetula* larvae due to the separate or combined application of *Metarhizium anisopliae* (M1cog) at two doses (H = 1 × 10^8^ or L = 1 × 10^6^ spores/insect) and 250 IJs of *Steinernema glaseri* (Sg) or *Steinernema carpocapsae* (Sc). SE, standard error.

The average mortality percentage of larvae of two sizes (Me and La) of *P. vetula* by the application of the fungus Ma in two doses (H or L) and the nematodes Sc and Sg during a 56 days period is presented in Figure 1. In treatments with only one entomopathogen, the highest larvae mortality was observed with MeSc (80 ± 11.5%) and no mortality was recorded in the LaH or MeL treatments. In combination treatments, the highest mortality was achieved in MeLSg (80 ± 11.5%) and the lower mortality was observed when the MeHSc (27 ± 17%) and LaHSc (27 ± 13%) treatments were applied. No larval mortality was observed in the untreated control (data not shown).

The statistical comparison of all treatments with respect to the control shows that only six treatments (Table 1) were statistically different (*p* < 0.05). In the combined treatments, the larger difference was observed with the MeLSg treatment (*p* = 0.002), followed by the MeHSg treatment (*p*  = 0.047) and the LaLSg and LaHSg ones. In the uncombined treatments, MeSc was the one with the greatest control difference (*p* = 0.002), while LaSg showed the minimum mortality difference. Five of the six better treatments applied included Sg.

**Table 1.** Results of the ANOVA analysis of means of each treatment compared with the untreated control (one degree of freedom).

Treatment	Mortality of *Phyllophaga vetula* (% ± SD)	*p*-value (ANOVA)
LaHSg	47 ± 13.3	0.031
LaLSg	60 ± 11.5	0.009
MeHSg	60 ± 20	0.047
MeLSg	80 ± 11.5	0.002
LaSg	40 ± 11.5	0.034
LaHSc	27 ± 13.3	0.116
LaLSc	33.3 ± 13.3	0.086
MeHSc	27 ± 17.6	0.219
MeLSc	47 ± 29	0.185
MeSc	80 ± 11.5	0.002
LaH	0	0
MeL	0	0

**Notes:** H, high dose of *Metarhizium anisopliae*; L, low dose of *Metarhizium anisopliae*; Sg, *Steinernema glaseri*; Sc, *Steinernema carpocapsae*; La, large-sized larvae; Me, medium-sized larvae.

The interaction analysis shows that the combined treatments LaHSg (47 ± 13.3%) and LaSg (40 ± 11.5%) presented a synergistic interaction between the fungus Ma and the nematode Sg, while an antagonistic one (*D* = −33.33) was observed between Ma and Sc.

### Effect of EPN on the mortality of *Phyllophaga vetula* larvae

**Figure 2:**
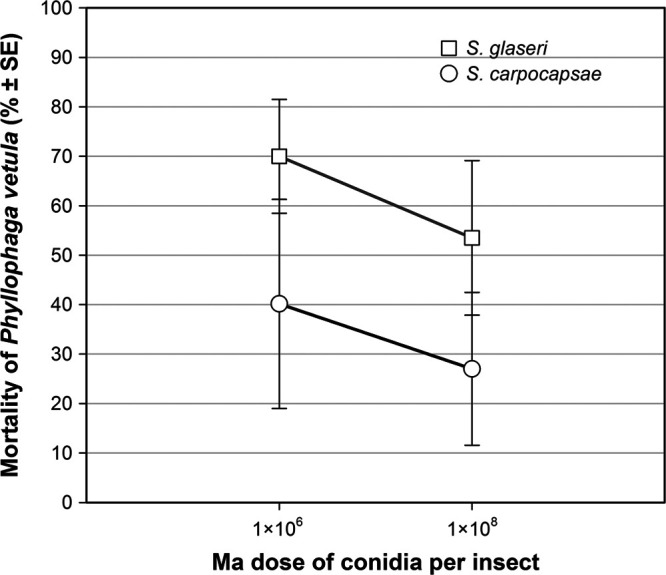
Mortality of *Phyllophaga vetula* due to *Metarhizium anisopliae* (M1cog) at two spore dosages, and two entomopathogenic nematode species. SE, standard error.

The ANOVA analysis between factors showed that the only significant difference was in the mean values among the different levels of the EPN factor and the comparison between Sg and Sc levels; Tukey tests showed that the difference of the two groups that were compared was statistically significant (*N* = 24, *q* = 3.438, *p* = 0.027). This difference can be seen in the graph of mean mortality for EPN by levels of Ma (Fig. 2), where the mortality percentage follows the same increasing trend in both levels of EPN with increasing doses of Ma, but without a significant influence of Ma on larvae mortality.

By analyzing the tested factors separately, it was found that the species of EPN applied was the most important component in the mortality percentage of *P. vetula*, with Sg being the nematode of best performance (*N* = 24, *q* = 3.438, *p* = 0.027). The factors that did not have a significant influence on the mortality percentage were insect size or dose of Ma. When the entomopathogens were combined, larvae mortality of size Me increased when the dose of Ma decreased, particularly in the combination Ma with Sc (Pearson *r* = −1.0, *p* = 0.0175).

### Effect of *Metarhizium anisopliae* dose on the mortality of *Phyllophaga vetula*

**Figure 3:**
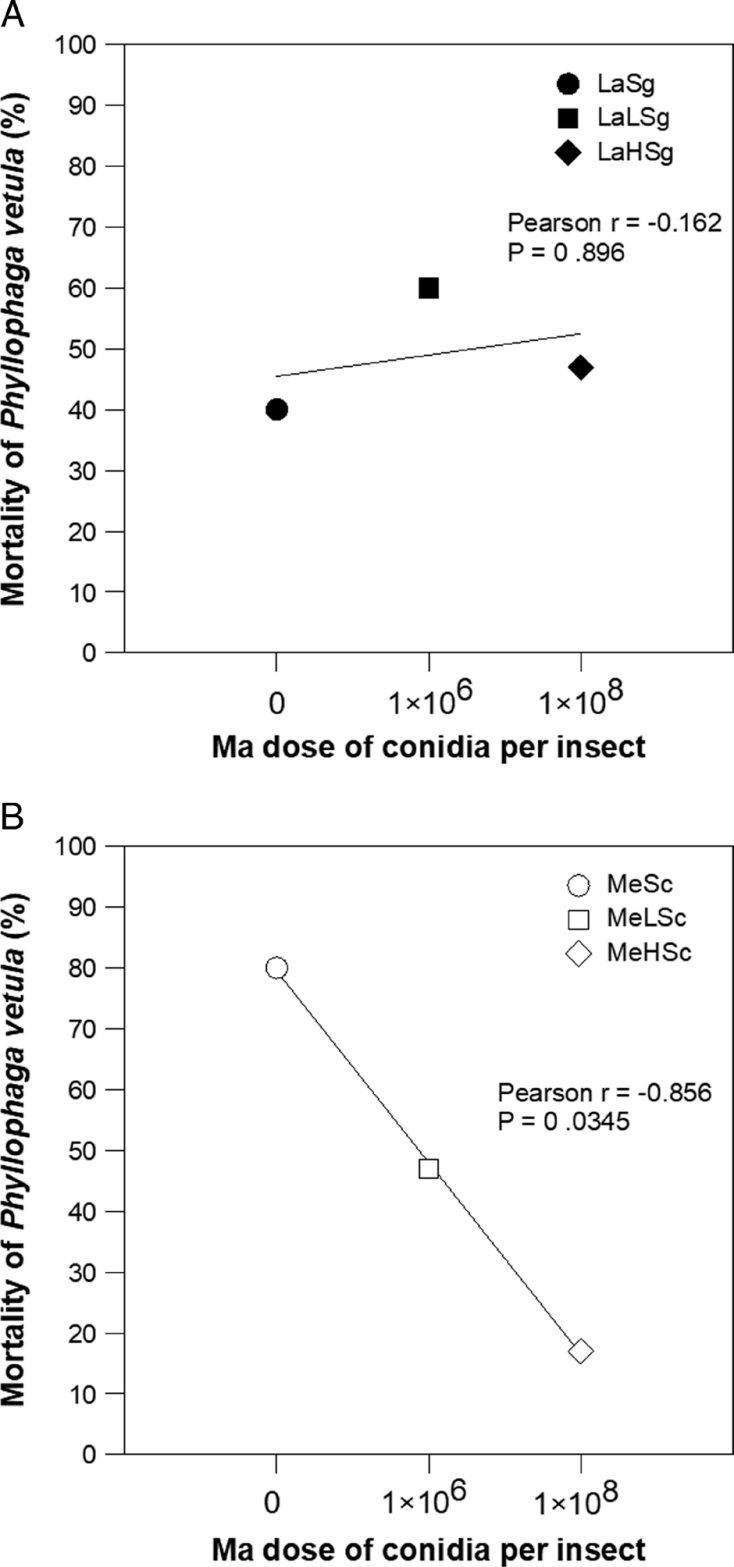
(A and B) Mortality of large (La) or medium (Me) *Phyllophaga vetula* larvae due to the increase in the dose of *Metarhizium anisopliae* M1cog, H = 1 × 10^8^ or L = 1 × 10^6^ spores/insect, before the application of 250 IJs of *Steinernema glaseri* (Sg) or *Steinernema carpocapsae* (Sc).

The mortality trend of two *P. vetula* larvae sizes due to the increase in the dose of Ma (H, L, and a control without fungus) before the treatment with Sg or Sc is shown separately. It can be seen that in the treatments with Sg (Fig. 3A), there was no significant relationship between dose and mortality of La larvae (Pearson *r* = −0.162, *p* = 0.896), whereas in the treatments with Sc the mortality of Me larvae tended to increased significantly (Pearson *r* = −0.856, *p* = 0.0345) when the dose of Ma decreased (Fig. 3B).

## Discussion

This is the first study to evaluate the mortality of *P. vetula* larvae of two sizes due to the separate or combined application of the fungus *M. anisopliae* and the nematodes *S. glaseri* or *S. carpocapsae*. In respect to separate applications, the results show that the mortality percentage by EPN went from regular to high, while no larvae died by the application of *M. anisopliae* alone. However, when the EPN and the fungus *M. anisopliae* were applied in combination, the interactions were of the additive and antagonistic types. The additive interaction happened between Ma and Sg and the insect mortality increased, whereas with the combined application of Ma and Sc, the interaction was antagonistic and insect mortality diminished.

The deferred application between EPF and EPN has been evaluated on third-instar larvae of *Phyllophaga polyphylla* by Martínez-Hernández et al. (2015) who reported an antagonistic effect and a low percentage of insect mortality. In that study, they first applied *Metarhizium pingshaense* (1 × 10^8^ conidia·mL^−1^) and six days later *H. bacteriophora* (50 IJs.mL−1), obtaining a mortality of 9.1 to 11.5% after 39 days. However, combining a *B. pseudobassiana* isolate (1 × 10^8^ conidia.mL−1) and one isolate of *H. bacteriophora* (100 IJs.mL−1), where the fungal pathogen was inoculated first, an additive interaction was identified.

The nematode Sc is usually not effective against larvae of *Phyllophaga* spp. This species has been tested at a dose of 5 × 10^9^ IJs.ha−1 against *P. anxia* larvae (LeConte) in Abeto Fraser tree production fields and no effective control was observed in relation to the control treatment over two years of the experiment, because it only killed 1 insect/m^2^ in the first year and less than 1 insect/m^2^ in the second year (Liesch and Williamson, 2010). When evaluated against third-instar larvae of *P. bicolor*, the nematode Sc (All strain) was slightly infective (22.92%), but it did not cause the death of any insect (Melo et al., 2010).

Third-instar larvae of *P. hirticula* were confronted to 24-h-old Sc at a concentration of 1,000 IJs/larva, but even though there were IJs on the body of the insect, the entry route was through the digestive tract (anus and mouth) and not through the spiracles or perforations in the cuticle (Forschler and Gardner, 1991), resulting in a lower percentage of penetration, and a low percentage of larvae mortality.

Sg is a nematode of greater effectiveness than Sc against some local species of *Phyllophaga* spp. In the laboratory, the dose of 1000 ± 50 IJs on third-instar larvae of *P. vetula* has achieved a mortality rate of 40 to 97.5% (Girón-Pablo et al., 2015; Cortés-Martínez et al., 2017), while Sc (All strain) had an intermediate performance because it required four days to control 50% of the larva when applied at doses of 200 IJs (Ruiz-Vega et al., 2003). In contrast, third-instar larvae of *P. crinita*, *P. congrua*, and *P. georgiana* were not very susceptible to Sg (NC strain) in doses of 400 IJs per insect, since the mortality percentage was 6 to 23% (Koppenhöfer et al., 2004). Only depending on application rate, Sc (All strain) combined with *B. bassiana* resulted in additive effect on *Curculio caryae* (Coleoptera: Curculionidae) (Shapiro-Ilan et al., 2004).

*M. anisopliae* is more effective than *B. bassiana* against *P. vetula*; the pathogenicity of HI-019 strain native of Morelos, Mexico, applied at a dose of 1 × 10^8^ conidia.mL−1 was 86% after 12 post-inoculation (Pérez et al., 2016). However, our study reports different results as *M. anisopliae* M1cog strain at doses of 1 × 10^6^ and 1 × 10^8^  spores/insect did not kill any larvae.

This result suggests that *M. anisopliae* M1cog strain tends to be a specialist rather than a generalist fungus, mainly because it is an isolate from *S. frugiperda*, which limits their ability to cause disease in multiple insects as Zimmermann (2007) and Wang et al. (2016) argued. Santi et al. (2010) pointed out that the insect cuticles induce the secretion of specific proteases to respond in a precise and specialized way to specific hosts.

On the basis of the results presented in this study and the hypothesis that suggests that the synergistic interactions strongly depend on the target host, pathogens that are combined and the application timing (simultaneous vs sequential) (Wakil et al., 2017), the combined application of an EPF and EPN showed no consistent effects on the mortality percentage of *P. vetula*, but certainly, additional exploratory studies in relation to these factors must be carried out to confirm this proposition.

## Conclusions

This study conducted under laboratory conditions showed that the separate application of EPN on *P. vetula* larvae caused mortalities of 40 and 80% using the nematodes *S. glaseri* (NJ-43 strain) and *S. carpocapsae* (All strain), respectively. However, the application of *M. anisopliae* (M1cog) did not cause any deaths because the fungus was isolated from *S. frugiperda*.

As for the combined application, an additive interaction between the time deferred application of the fungus *M.* a*nisopliae* and *S. glaseri* caused a slight increase in mortality from 40 to 47% on *P. vetula* large-sized larvae, while the antagonistic effect by the deferred application of *M. anisopliae* and *S. carpocapsae* significantly diminished its mortality percentage from 80 to 27% on medium-sized larvae.
